# Non-Coding RNAs in Diagnostic Pathology of High-Grade Central Osteosarcoma

**DOI:** 10.3390/diagnostics15111355

**Published:** 2025-05-28

**Authors:** Albert Roessner, Sabine Franke, Julian Schreier, Sarah R. Ullmann, Franziska S. Karras

**Affiliations:** Institute of Pathology, Otto-von-Guericke University Magdeburg, Leipziger Str. 44, 39120 Magdeburg, Germany; sabine.franke@med.ovgu.de (S.F.); jschreier03@yahoo.de (J.S.); sarah.ullmann@orthoclin.de (S.R.U.); franziska.karras@med.ovgu.de (F.S.K.)

**Keywords:** highly malignant osteosarcoma, non-coding RNAs, differential diagnosis

## Abstract

A histological evaluation remains the cornerstone of diagnosing highly malignant osteosarcoma, having demonstrated its efficacy and reliability over several decades. However, despite these advancements, misdiagnoses with severe consequences, including inadequate surgical procedures, continue to occur. Consequently, there is a pressing need to further enhance diagnostic security. Adjunct immunohistochemical approaches have demonstrated significant effectiveness in regard to cancer diagnostics, generally. However, their utility for identifying highly malignant osteosarcoma is limited. Molecular genetic findings have significantly improved the diagnosis of Ewing’s sarcoma by identifying specific translocations and have been used to detect specific *IDH* gene mutations in chondrosarcoma. Nevertheless, molecular genetic alterations in highly malignant osteosarcoma exhibit a high degree of complexity, thereby limiting their diagnostic utility. Given that only 1–2% of the human genome comprises protein-coding sequences, the growing number of non-coding regulatory RNAs, which are increasingly being elucidated, has garnered substantial attention in the field of clinical cancer diagnostics. Over the past several years, patterns of altered non-coding RNA expression have been identified that facilitate the distinction between benign and malignant tumors in various organs. In the field of bone tumors, the experience of this approach has been limited thus far. The divergent expression of microRNAs has demonstrated utility for differentiating osteosarcoma from osteoblastoma and discriminating between osteosarcoma and giant-cell tumors of bone and fibrous dysplasia. However, the application of non-coding RNA expression patterns for the differential diagnosis of osteosarcoma is still in its preliminary stages. This review provides an overview of the current status of non-coding RNAs in osteosarcoma diagnostics, in conjunction with a histological evaluation. The potential of this approach is discussed comprehensively.

## 1. Introduction

High-grade central osteosarcoma is the most common malignant bone tumor and the most common entity of osteosarcomas. Histologically, it has to be thoroughly distinguished from the other types of osteosarcomas [1]. It is predominantly observed during the second decade of life, although there is a secondary peak in individuals over the age of 40 [2]. In older patients, the efficacy of chemotherapy is reduced [3]. Males are more commonly affected. The preferred sites within the skeleton are the distal femur, proximal tibia, and proximal humerus, although other locations in long bones can also occur. Osteosarcomas of the jaw are rare and have to be assessed differently [4].

The primary symptom is usually pain in the affected region. The interval between the onset of the first clinical symptoms and the diagnosis ranges from weeks to several months, with prolonged courses being uncommon. The prognosis of high-grade osteosarcoma has significantly improved since the 1970s, through the use of combined neoadjuvant chemotherapy with methotrexate, doxorubicin, cisplatin, and under certain conditions, ifosfamide [5]. With surgical treatment alone, the 5-year survival rate was 10–20%, according to all major statistics. However, with the introduction of neoadjuvant chemotherapy, this rate has increased to 65–70% [6,7]. However, this improvement only applies to patients with localized tumors. The 5-year survival rate remains consistently poor, at approximately 30%, for patients with metastatic osteosarcoma, even after treatment with combined chemotherapy [7]. The therapeutic effect of preoperative chemotherapy can be effectively assessed histologically using the tumor resection specimen [8]. More details in this regard are provided in chapter 9. However, a correlation between the therapeutic effect and the histological subtype remains questionable [9]. From an oncological perspective, it is highly unsatisfactory that a plateau phase in the treatment of osteosarcoma patients has been reached in regard to conventional chemotherapy for approximately 40 years. Evidently, no further advancements can be expected from conventional chemotherapy approaches. Similarly, no fundamental developments in the understanding of high-grade osteosarcoma can be anticipated, based on histology and immunohistology alone [10]. Recent comprehensive reviews of osteoblastic bone tumors increasingly include molecular genetical and molecular pathological aspects [11,12].

Radiologically, the tumor originates centrally, rapidly destroying the cortex and invading adjacent soft tissues. If the tumor forms abundant mature bone, the radiographic image appears sclerotic. If unmineralized tissue predominates, the tumor presents as an osteolytic type. Macroscopically, highly malignant osteosarcoma involves the metaphyseal region, often extending into soft tissues. Epiphyseal involvement is rare, occurring in less than 5% of patients [13]. Some progress has been made over the past several years in regard to the primary diagnosis of highly malignant osteosarcoma in the field of radiology and, to a lesser extent, in the field of histopathology [1]. Molecular genetic studies of osteosarcoma have revealed tumors of high molecular complexity, yet they lack specificity, rendering them of limited utility for primary diagnostics [14]. In light of this unsatisfactory situation, ncRNAs have emerged as a promising area of focus to address differential diagnostic challenges in regard to highly malignant osteosarcoma. Consequently, this review focuses on the potential of ncRNAs as an adjunct to a primary histological diagnosis, which has been the leading therapy until now [15]. To identify pertinent and specialized literature, a comprehensive search was conducted in the PubMed and Web of Science databases, employing a range of subject headings to specifically focus on the diagnosis of highly malignant osteosarcoma utilizing non-coding RNAs (ncRNAs). This strategy excluded the extensive literature on general aspects of ncRNAs in osteosarcoma, such as prognosis and therapeutic strategies.

## 2. Histologic Characteristics

**Figure 1:**
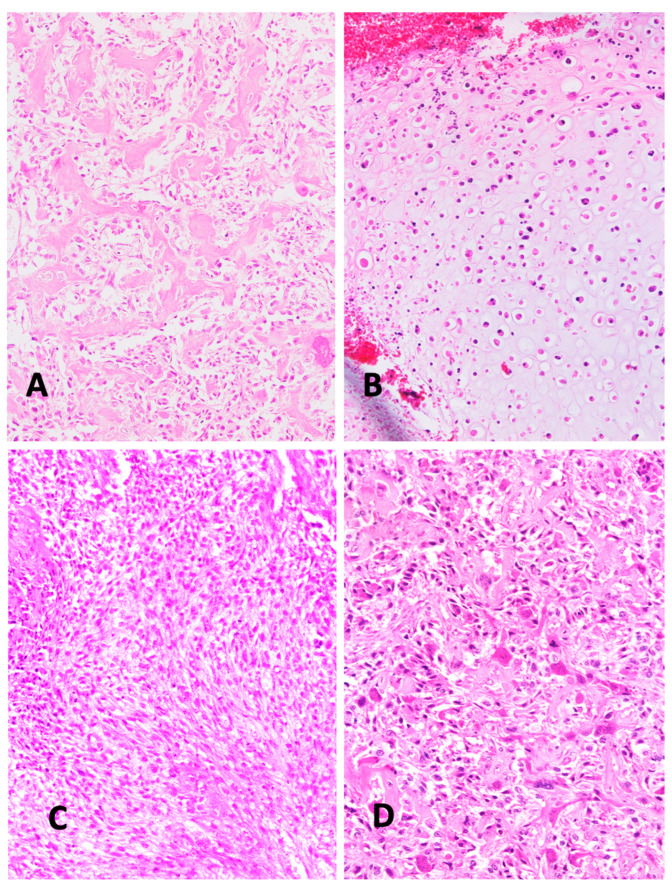
(**A**) Typical osteoblastic osteosarcoma, with ample osteoid formation appearing as broad homogenously stained trabeculae (H&E ×200). (**B**) Chondroblastic osteosarcoma, with obvious cartilage nature of the sarcoma tissue (H&E ×200) (**C**) Fibroblastic osteosarcoma, with typical fibroblast-like spindle cells. This pattern resembles connective tissue. Its malignant nature is revealed by nuclear pleomorphism and mitoses (H&E ×200). (**D**) Giant cell–rich osteosarcoma, containing abundant osteoclast-like giant cells, with multiple regularly formed nuclei (H&E ×200). (All histological images presented in this manuscript originate from the senior author’s personal archive).

Various subtypes of osteosarcoma can be distinguished. In osteoblastic osteosarcoma, tumor cells predominantly produce osteoid (Figure 1A). The chondroblastic variant is characterized by neoplastic cartilage (Figure 1B), while the fibroblastic type consists of highly malignant spindle-shaped mesenchymal tumor cells (Figure 1C). Tumor cells consistently exhibit severe nuclear atypia and increased mitotic activity. Rare subtypes include the giant cell–rich variant (Figure 1D). Telangiectatic osteosarcoma can appear to be similar to aneurysmal bone cysts. However, higher magnification reveals increased mitotic activity and atypical mitoses. The diagnosis of osteosarcoma always requires the detection of at least some osteoid-producing tumor cells. The ability of mesenchymal tumor cells to produce osteoid is considered fundamental to their biological and oncological behavior [16]. The diagnosis of osteosarcoma cannot be made if no tumor osteoid is identified in a malignant bone tumor. Since multifold tissue differentiations can often be found within the same osteosarcoma, the histological appearance of high-grade intramedullary osteosarcoma is extremely heterogeneous. This heterogeneity has raised questions about whether different histological types indicate different prognoses. So far, studies have not definitively shown that osteoblastic, chondroblastic, fibroblastic, telangiectatic, or giant cell–rich osteosarcomas have distinct prognoses [17]. A more recent study suggests that a histological scoring system may have some prognostic relevance [18]. This scoring system, however, focuses less on histological subtypes and more on characteristics indicative of malignancy, such as the number of mitoses and the extent of vascular invasion.

## 3. Molecular Genetic Characteristics

Extensive alterations in the p53 gene have been comprehensively documented in osteosarcoma, commencing in 1987 [19]. Changes in the *RB* gene were also identified in early research [20]. The profound significance of molecular genetic alterations in the pathogenesis of high-grade osteosarcoma was only fully comprehended after the advent of next-generation sequencing (NGS). A pioneering comprehensive study in this domain was conducted by Chen et al. [21], followed by additional studies from Behjati et al. [22], Bousquet et al. [23], Chiappetta et al. [24], Kovac et al. [25], and Perry et al. [26]. In contrast to the majority of malignant tumors, in which single nucleotide variations (SNVs) constitute the bulk of the genetic alterations [27,28,29], in osteosarcoma, structural variations (SVs) and copy number variations (CNVs) are predominant [12]. Comparative molecular genetic studies of other malignant pediatric tumors have revealed that juvenile highly malignant osteosarcomas exhibit the highest prevalence of structural variations among all pediatric tumors [30]. A specific mechanism of dramatic chromosomal alterations is chromothripsis, derived from the Greek words, “chromos”, meaning chromosome, and “thripsis”, meaning shattering. This genetic phenomenon was initially described by Stephens et al., in 2011 [31]. In contrast to the gradual model of the adenoma-carcinoma sequence, chromothripsis generates hundreds of genetic alterations in a single cellular crisis, involving one or more chromosomes. This phenomenon is observed in only 2–3% of all malignant tumors, but in up to 30% of juvenile osteosarcomas [23]. Another genetic alteration phenomenon is kataegis (Greek for “thunderstorm”). This phenomenon involves hypermutated regions, with distinctive characteristics, on chromosomes. It was initially described in breast cancer [32,33]. Kataegis is also found in osteosarcoma [22].

Genomic alterations and allelic imbalances have been proposed as prognostic indicators in highly malignant osteosarcoma [34]. Molecular genetic alterations in this type of tumor generally exhibit a high degree of complexity, which limits their diagnostic utility to date [14]. This is in contrast to chondrosarcoma, where *IDH* mutations have been used as a useful diagnostic tool [35] and specific translocations have been identified in Ewing’s sarcoma [33]. Genomic sequencing of osteosarcoma cases has revealed distinct genetic alterations that may serve as the foundation for future targeted therapy interventions [36].

## 4. Challenges in Regard to the Differential Diagnosis of Highly Malignant Osteosarcoma

**Figure 2:**
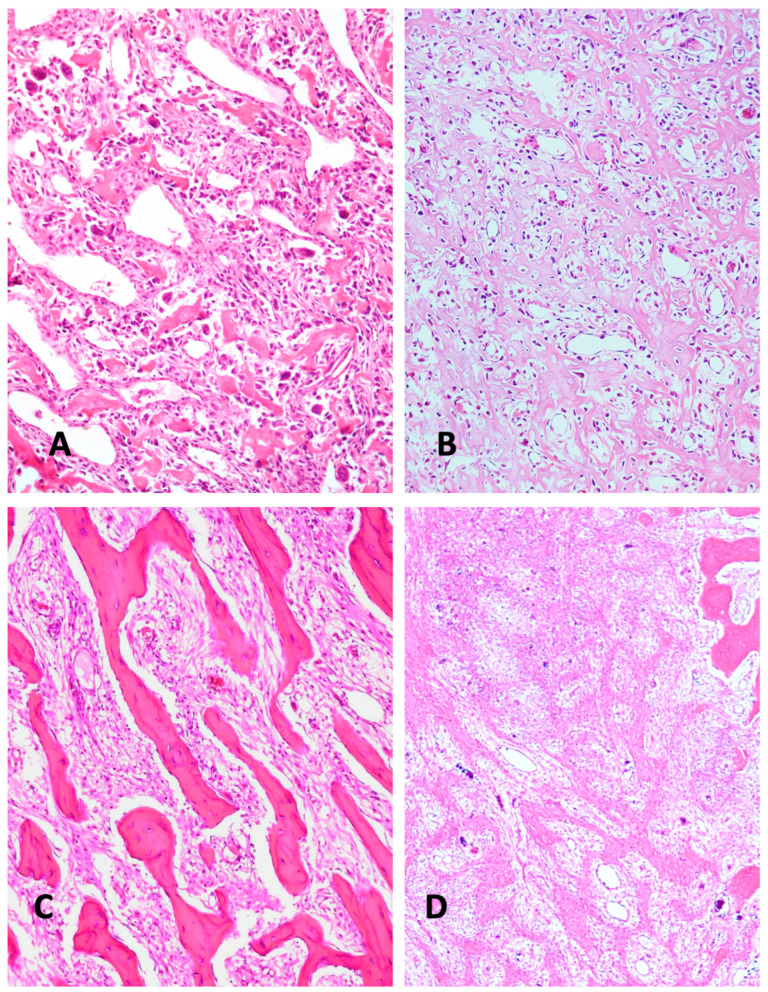
(**A**) Aggressive osteoblastoma, with atypical cellular nuclei. The pronounced vascularization is typical of osteoblastoma (H&E ×200). (**B**) Highly malignant osteoblastic osteosarcoma, with a high level of nuclear pleomorphism and typical osteoid formation, indicted by the tumor cells appearing as broad trabeculae (H&E ×200). (**C**) Low-grade intramedullary osteosarcoma. In this typical case, the bone structure is very highly differentiated and barely discernable from reactive bone formation (H&E ×200). (**D**) Heterotopic ossification mimicking osteosarcoma. In particular, the immature bone trabeculae are very similar to osteosarcoma (H&E ×200).

**Figure 3:**
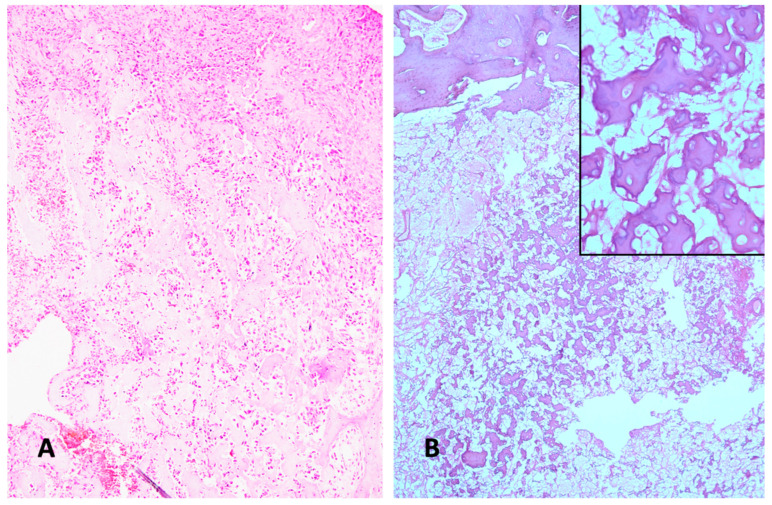
(**A**) Osteoblastic osteosarcoma before preoperative chemotherapy. Viable malignant tumor cells, lacking any signs of regression or necrosis. Note the well-stained nuclei of the viable tumor cells (H&E ×100). (**B**) Completely devitalized necrotic area of the former osteoblastic osteosarcoma, with no viable tumor cells left after preoperative chemotherapy. In sharp contrast to the preoperative tissue, no stained nuclei can be observed (H&E ×100). Inset: high-power view of completely devitalized former osteoblastic tumor tissue and remnants of an osteoid. Not one single viable tumor cell is left (H&E ×400).

**Figure 4:**
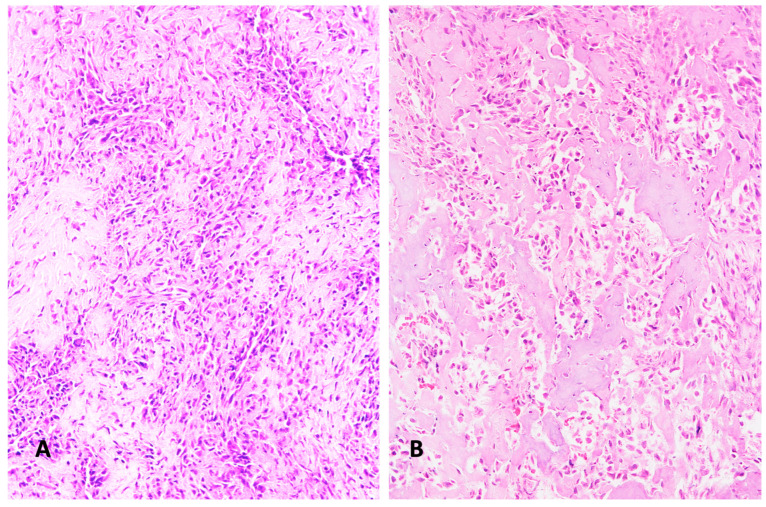
(**A**) Typical histological appearance of primary osteoblastic osteosarcoma. The patient did not develop lung metastases. The pleomorphic tumor cells reveal its malignant nature (H&E ×200). (**B**) Primary osteoblastic osteosarcoma, with lung metastasis, at the time of diagnosis (H&E ×200). The non-metastasizing and metastasizing malignant tumors appear histologically indistinguishable.

The differential diagnosis of other bone tumors and lesions primarily includes osteoblastoma (Figure 2A,B). Additionally, giant cell-containing osteosarcoma (Figure 1D) must be differentiated from giant-cell tumors of bone and from chondroblastoma. Undifferentiated pleomorphic sarcoma-like osteosarcoma is barely distinguishable from undifferentiated pleomorphic sarcoma when an osteoid is lacking in the former. Another challenge in terms of differential diagnosis that occurs sometimes concerns discriminating between an aneurysmal bone cyst and telangiectatic osteosarcoma. Low-grade central osteosarcoma is also included in the differential diagnostic spectrum (Figure 2C). Even reactive lesion heterotopic ossification can, in the proliferative phase, cause differential diagnostic problems (Figure 2D). All these lesions require a therapy that is completely different from that given to osteosarcoma patients. Another notable histodiagnostic challenge concerns the prediction of the chemotherapy response (Figure 3) and, subsequently, the prediction of metastatic risk (Figure 4). Although the histology of highly malignant osteosarcoma has been meticulously described over several decades, the prognostic histologic indicators have never been convincingly validated.

The potential of molecular genetics to contribute to resolving the differential diagnostic challenges associated with bone tumors has been comprehensively addressed in numerous scholarly publications [37,38,39]. Furthermore, numerous articles on ncRNAs and osteosarcoma have been published in the past decade [15,40,41,42]. The majority of these articles are more focused on prognosis and general diagnostic markers [43] rather than on primary tumor diagnosis in relation to their histological appearance, which remains the essential and legally relevant basis for initiating a specific therapy for osteosarcoma patients. Consequently, this article concentrates on those ncRNAs that can be beneficial in regard to solving the differential diagnostic challenges concerning highly malignant osteosarcoma. It is crucial to emphasize that a precise diagnosis is paramount for guiding patient therapy and ensuring patient survival [1].

## 5. The Use of ncRNAs in Translational Biology

**Figure 5:**
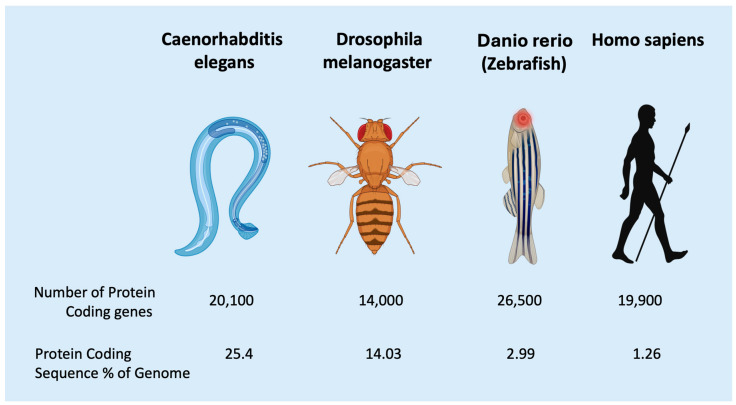
There is no correlation between the number of protein-coding genes and the developmental complexity of a species. Instead, there is a negative correlation between the percentage of the genome occupied by protein-coding sequences and developmental complexity [67] (Created with BioRender).

It has become increasingly evident that only 1–2% of the human genome’s coding sequence encodes for proteins [44] (Figure 5). In addition to the RNAs with coding potential, there are substantial quantities of RNAs that lack coding potential [45]. The latest edition of the human genome catalog posits that the human genome comprises approximately 20,000 protein-coding genes. This figure has been steadily declining since the 1980s, when it was estimated to be over 100,000 genes [46]. Consequently, we now understand that coding genes constitute only a minuscule fraction of the human genome [47]. Remarkably, this toolkit of protein-coding genes has remained essentially unchanged since the early stages of metazoan evolution, even in sponges that appeared more than 600 million years ago [48].

The human genome also contains hundreds of thousands of regulatory elements that do not encode proteins. Previously, these elements were dismissed as “junk DNA” [49,50,51,52]. In contrast to the misconceived hypothesis of “junk DNA”, the recognition that ncRNAs perform crucial biological functions has been hailed as a major paradigm shift in contemporary molecular biology [53,54]. The role of messenger RNA (mRNA), transfer RNA (tRNA), and ribosomal RNA (rRNA) in gene expression was established in the 1950s. However, it was not until the end of the 1990s that the discovery of microRNAs (miRNAs) and several other small ncRNAs, along with their pivotal roles in the post-transcriptional regulation of gene expression, particularly in eukaryotic organisms, gained widespread recognition [55,56].

### 5.1. The Functions of Regulatory ncRNAs in Regard to Metazoan Differentiation

It has long been observed that the amount of ncRNAs increases with developmental complexity, assessed in regard to the increasing number of differentiated cell types [57,58]. On top of that, ncRNAs also play a central role in human development and cognition [59]. In addition to other factors, such as distal enhancers and transcription factors, regulatory ncRNAs enable the regulation of temporal and spatial gene expression in evolutionary processes, which is a precondition for increasingly complex multicellularity in higher metazoan organisms [60]. Alternative splicing was not considered to be a sufficient biological strategy for increasing the biodiversity in the metazoan world [61]. This is also corroborated by the observation that the number of protein-coding genes in the genome has remained relatively divergent and did not constantly increase throughout metazoan evolution, from simple organisms, such as C. elegans, to homo sapiens [62,63] (Figure 5). The number of protein-coding genes does not directly correlate with the organism’s complexity or the number of differentiated cell types. Conversely, the decreasing ratio of protein-coding sequences in regard to its percentage of the entire genomic DNA does [57]. Given the pivotal role of ncRNAs in cellular differentiation, it is plausible to presume that they exhibit a correlation with histological diagnostics, which predominantly concentrate on cellular differentiation [64,65]. Among the various classes of ncRNAs, the role of microRNAs has been the subject of the most extensive investigation to date [66].

### 5.2. Classification of ncRNAs, Basic Facts

Non-coding RNAs (ncRNAs) are primarily categorized into two distinct classes, based on the number of nucleotides. Small non-coding RNAs (sncRNAs) are defined as molecules with a length of less than 200 nucleotides, while long non-coding RNAs (lncRNAs) exceed 200 nucleotides in length [45]. In the context of tumor diagnostics, sncRNAs and lncRNAs, including circular RNAs (circRNAs) have the most practical importance. Consequently, this review focuses on these types of ncRNAs [68,69,70]. Within the class of sncRNAs, microRNAs (miRNAs) have garnered the most extensive research attention in the field of cancer. Their primary function is to cause the negative regulation of gene expression by targeting specific messenger RNAs, leading to their dysfunction and degradation [71,72].

The details of miRNA biogenesis are discussed elsewhere [64].

## 6. The Use of ncRNAs as Diagnostic Biomarkers in Cancer

An ongoing debate centers on whether the classification of human tumors, based on their tissue of origin, remains pertinent in the context of cancer genomics and precision oncology [65]. Historically, a histologic evaluation of tissue biopsies, augmented by immunohistochemistry, has been the cornerstone of definitive cancer diagnosis [73,74]. In regard to the majority of tumor diagnoses, alternative diagnostic methods that can be utilized essentially have an adjunctive role. This is the case for image radiology and other conventional methods used in laboratory medicine. Molecular genetic methods centered on the whole genome or whole exome NGS have been demonstrated to significantly enhance histopathologic diagnoses in approximately 15% of all cancer diagnoses [75]. Prior to this background, therapeutic approaches that are agnostic with regard to histology are still the subject of ongoing debate [76]. Until now, the basic principle of histopathologic tumor classifications has been the evaluation of tumor tissue in relation to its tissue of origin and the degree of similarity to the tissue of origin [77]. It can be assumed that the future potential of liquid biopsies is not only powered by the analysis of circulating-free tumor DNA (cfDNA), but also by the analysis of different classes of ncRNAs.

The extensive regulatory RNA machinery is responsible for the evolution of metazoan complexity, with increasingly specialized cell types that are contingent upon the evolutionary stage [78]. It can be assumed that the differential expression of non-regulatory RNAs corresponds to the histopathological tumor classification and diagnosis [79,80,81]. In the past several years, extensive reviews have been published on the central role of ncRNAs in cancer [82,83,84]. A main advantage of ncRNAs as a tool for cancer diagnostics and classification is their detectability in plasma, serum, and other body fluids [85,86]. This is all the more important, because the risk of tumor cell seeding through biopsies cannot be entirely ruled out [87]. In particular, exosomes as carriers of different ncRNAs are considered to be an important diagnostic tool [88,89]. Liquid biopsies have also demonstrated remarkable success in detecting gene alterations in cancer patients [90,91].

### 6.1. The Use of miRNAs as Tools in Cancer Diagnosis

The biogenesis and mechanism of action of miRNAs have been elucidated in the past several years [64]. The utility of miRNA patterns in the diagnosis of cancer has long been a subject of discussion. Their use as a diagnostic tool is evident, because these small molecules show stability in different fluids in the human body [78,92,93]. For example, miRNAs are particularly useful as a biomarker for breast cancer diagnosis [94]. In addition to cancer diagnosis, miRNAs also have the potential to predict drug efficiency and the clinical prognosis of cancer patients [68]. They show a general downregulation in tumors. Poorly differentiated tumors can also be successfully classified using miRNAs [95,96]. To date, a correct histopathological diagnosis remains the basis for discriminating between benign and malignant tumors. However, uncountable cases of so-called “borderline tumors” in the field of bone and soft tissue tumors [97] point to the limitations of the many-decades-old histopathologic approach in regard to establishing the malignancy of tumors. Recently, it was shown that comprehensive miRNA expression profiles, combined with a computational deep cancer classifier, were able to differentiate between breast cancer and skin cancer and their benign histologic counterparts. This might be considered to be the beginning of the use of computational classifiers for identifying the malignant traits of a given tumor [98].

### 6.2. The Use of lncRNAs as Diagnostic Biomarkers in Cancer

Over the past several years, it has become increasingly apparent that a significant portion of the human genome is transcribed into a multitude of long non-coding RNAs (lncRNAs). The classification and function of lncRNAs have been extensively described in numerous publications [99,100]. Moreover, lncRNAs have been shown to have functions in many molecular and cellular processes, as well as in development [101]. They have an important role in cancer pathogenesis [102]. Additionally, they exhibit tissue- and tumor-specific expression patterns [103].

### 6.3. The Use of circRNAs as Diagnostic Biomarkers in Cancer

Specifically, circRNAs, characterized by their covalently closed ring-like structures, exhibit exceptional chemical stability and demonstrate remarkable resistance to the activities of ribonucleases, due to the absence of free ends. These unique properties make them promising diagnostic and prognostic markers of cancer [104]. The first endogenous human circRNAs were identified in 1991. A comprehensive timetable depicting the discovery and development of knowledge on circRNAs in the field of cancer is provided by Pisignano et al. [105]. Their considerable value in molecular cancer diagnosis has increasingly been emphasized by others [106]. For example, it has been convincingly shown that three specific circular RNAs in serum exosomes were successfully applied as diagnostic biomarkers for non-small-cell lung cancer in the Chinese population [107], and a specific exosomal serum circRNA could serve as a diagnostic biomarker for colorectal cancer [108]. However, it is recommended that larger and more controlled clinical studies take place before applying circRNAs as secure diagnostic and therapy-guiding factors in clinical oncological practice [109].

### 6.4. The Utility of ncRNAs in Differentiating Between Benign and Malignant Tumors

Table 1 presents examples of the successful application of ncRNAs for discriminating between benign and malignant tumors in different organs. This approach is also applicable to the skeletal system, wherein miRNAs are useful in distinguishing enchondroma from low-grade chondrosarcoma [110]. In other organs, miRNAs can be used to discriminate between benign prostatic hyperplasia and prostatic cancer [111]. MiRNAs, particularly miRNA-122, have been validated for discriminating between thyroid cancer and benign nodules [112]. In breast cancer patients, it is possible to discriminate between early stages of breast cancer and benign diseases [113]. In another study, circulating miRNAs demonstrated their capacity to detect breast cancer in comparison to high-risk benign breast tumors [114]. A panel of potential lncRNA biomarkers was identified as being useful for distinguishing between benign and malignant liver tumors [115]. In a landmark study, Kaczmarek et al. applied a deep cancer classifier to discriminate between neoplastic tissue and non-neoplastic tissue on the basis of differential miRNA expression, focusing on non-neoplastic tissue and breast cancer and non-neoplastic tissue and skin cancer [94]. Defining different miRNA panels can also be useful in discriminating between benign and malignant pleura effusions [116]. Distinguishing between malignant borderline tumors and malignant ovarian cancers, solely based on histological findings, presents a diagnostic challenge. Moreover, miRNAs have also been proven to be valuable in regard to this type of differential diagnosis [117]. A notable challenge in regard to histopathological diagnosis lies in the distinction between adrenocortical adenoma and carcinoma. In this context, miRNA profiles can serve as a valuable supplementary tool for making this distinction [118].

## 7. The Utilization of Non-Coding RNAs as a Complementary Approach to the Histological Differential Diagnosis of Highly Malignant Osteosarcoma 

Even today, highly malignant osteosarcoma may be misdiagnosed as another tumor entity, resulting in inappropriate treatment, including the wrong surgical procedures [119]. Osteoblastoma is typically characterized radiologically as a well-defined, circumscribed lesion that does not present diagnostic challenges in standard clinical scenarios. However, sometimes diagnostic problems can arise in regard to discriminating between osteoblastoma and osteosarcoma. This is particularly the case for aggressive osteoblastoma and so-called epithelioid osteoblastoma, wherein atypical nuclei may cause some diagnostic confusion [120]. Furthermore, a tumor entity of osteoblastoma that is like osteosarcoma has been established, which can generate differential diagnostic problems in both directions [121]. Recently, recurrent translocations in *FOS* and *FOSB* have been detected in osteoblastoma, as well as osteoid osteoma, and may be of diagnostic value [122,123]. However, osteosarcomas with FOS expression have rarely been described [124]. Furthermore, methylation and copy number profiling might be useful for differentiating osteoblastoma from malignant tumors [125]. In the study by Riester et al. [126], miRNAs from FFPE tumor specimens of 11 osteoblastomas and 11 osteosarcomas were extracted and analyzed using high-throughput miRNA sequencing. The elevated expression of hypoxia-related miRNA-210 in the osteosarcoma cases in comparison to the osteoblastoma cases may be a future diagnostic adjunct in discriminating between osteoblastoma and osteosarcoma. In addition to this study, investigations of ncRNAs of osteoblastoma are very rare and do not mention differential diagnostic or biomarker aspects [127]. So far, there has only been one study published on the differential diagnosis of giant-cell tumors of bone and osteosarcoma [43]. The few other available studies on giant-cell tumors of bone refer to lncRNA expression in regard to the recurrence of giant-cell tumors [128] or general aspects of miRNA expression [127,129]. Araki et al. [43] found that patients with osteosarcoma have an increased serum level of miR-1261, not only compared to patients with giant-cell tumors of bone, but also to patients with fibrous dysplasia, osteoblastoma, and chondrosarcoma. No substantial research studies on ncRNAs in chondroblastoma have been published. Similarly, no research studies have been conducted on ncRNAs in aneurysmal bone cysts, so far.

Even the reactive lesions of traumatic heterotopic ossification (THO) can pose diagnostic challenges in regard to the differential diagnosis of osteosarcoma [1]. A recent study of miRNAs in THO could contribute to a better understanding of the underlying mechanisms and offer new possibilities for therapeutic targets [130]. However, the differential diagnostic aspects are not yet available. Mierzejewskiy et al. [131] showed that miR-99b, miR-146, miR-204, and LINC00320 were upregulated in THO, when compared with normal bone and muscle tissue. In future, these ncRNAs might serve as useful biomarkers for the differential diagnosis of THO from highly malignant osteosarcoma (Figure 2D).

In summary, the analyses of various ncRNA categories have thus far yielded only limited reliable data to assist histological diagnoses in distinguishing between highly malignant osteosarcomas, benign tumors, reactive lesions, and low malignant osteosarcomas (Table 2). In contrast, there are numerous results available for discriminating between malignant tumors and benign lesions in cancers of other organs (Table 1). Consequently, there is an urgent need to apply advanced molecular data from the field of ncRNAs to enhance the differential diagnoses around osteosarcoma to a more effective level.

## 8. The Utilization of Non-Coding RNAs as Comprehensive Diagnostic Biomarkers for Highly Malignant Osteosarcoma 

The ncRNAs in serum or plasma can be useful as diagnostic markers for the early detection of osteosarcoma, as has been extensively discussed by Araki et al. [43]. This feature can also facilitate the primary diagnosis of osteosarcomas, even before taking biopsies. Other studies also focus on the utility of ncRNAs as prognostic markers. Since this review centers on the diagnosis of osteosarcoma, Table 3 depicts the most important markers for early primary diagnosis. Studies that focus on prognostic and therapeutic aspects are not considered here.

## 9. The Potential of Non-Coding RNAs in Predicting the Chemotherapy Response 

Since the advent of neoadjuvant chemotherapy for osteosarcoma patients, histological investigations of post-chemotherapy operation specimens have been of considerable oncological interest. These investigations have been employed to assess the extent of regression alterations and tumor necrosis associated with the chemotherapy effect [141,142]. The ratio of necrosis in relation to viable tumor tissue with at least 90% necrosis has been considered to be a prognostic factor in the majority of studies, correlating with the patient’s outcome [143]. However, this general experience has not been substantiated. A multivariate analysis confirmed the prognostic significance of the patient’s age and disease stage, while the poor necrosis rates did not reach statistical significance [144].

This implies that the ratio of necrosis in post-chemotherapy specimens cannot be reliably utilized as a definitive factor for guiding patient therapy. Deep learning-based analysis of tumor resection specimens did enhance the accuracy of the histologic investigation, but did not enhance the prognostic value [145]. Whole-exome sequencing genomic analysis revealed only slight variations between histologic responders and non-responders among osteosarcoma patients, indicating that this methodical approach has not attained unequivocal clinical significance so far [24]. Advanced radiological strategies can provide some indications of the chemotherapy response in patients, but they cannot be considered sufficiently reliable for making therapy-related decisions [146,147,148]. A comprehensive evaluation of coding gene expression through the analysis of mRNA expression profiles, in conjunction with lncRNAs, appears to have significant value. Nevertheless, the clinical applicability of this approach as a diagnostic tool in oncology remains restricted, so far [149].

Given the aforementioned background, the role of ncRNAs has been discussed as a novel and effective tool for predicting the chemotherapy response in osteosarcoma patients for several years. The neoadjuvant chemotherapy regimen for osteosarcoma patients has traditionally been based on the combination of high-dose methotrexate (HD-MTX), Adriamycin (ADR), and cisplatin (DDP) [150], with the possible addition of ifosfamide for poor responders and patients with metastases at presentation [151]. The interplay of different classes of ncRNAs with the pharmacological and cytotoxic effects of these drugs and on multidrug resistance (MDR) is a major topic in current osteosarcoma research [152]. The number of publications exploring the role of ncRNAs in regard to the chemotherapy effects on osteosarcoma has surged significantly over the past few years. While many of these studies are conducted in vitro, utilizing established single-cell lines, their practical clinical relevance may be limited. In contrast, in vivo studies on human tumor tissue or body fluids are considerably rarer. In the following section, a concise overview of cell culture studies is provided, with the in vivo studies discussed in greater detail.

### 9.1. Cell Culture Studies

The miRNA-29 family has a tumor suppressor role in regard to methotrexate resistance and can promote cell apoptosis [153]. Regarding the effects of ncRNAs on cisplatin, it was discovered that a knockdown of lncRNA ANRIL enhances osteosarcoma cells’ sensitivity to cisplatin-induced cytotoxicity. This finding has prompted speculation regarding ANRIL as a potential therapeutic target for osteosarcoma chemotherapy [154]. The lncRNA GAS5 promotes cisplatin chemosensitivity via the GAS5/miR-26b-5p/TP53INP1 axis, pointing to lncRNA GAS5 as a possible indicator for cisplatin-based chemotherapy [155]. Furthermore, it has been demonstrated that circRNA CircUBAP2 plays a pivotal role in the cisplatin resistance of osteosarcoma cells by modulating the expression of miR-506-3p [156]. Circ-RNA CHI3L levels were increased in cisplatin-resistant osteosarcoma cells and circRNA-CHI3L1.2 knockdown sensitized cisplatin-resistant osteosarcoma cells to cisplatin through the miR-340-5p–LPAATβ axis [157]. The lncRNA HOTAIR was shown to promote the cisplatin resistance of Saos2/DDP, MG-63/DDP, and U2OS/DDP cells by affecting cell proliferation, invasion, and apoptosis via the miR-106a-5p/STAT3 axis [158]. Numerous cell culture studies have underscored the significance of ncRNAs in mediating the diverse effects of doxorubicin. For instance, miRNA-150 has the ability to sensitize osteosarcoma cells to chemotherapy treatment with doxorubicin [159]. The overexpression of miR-506-3p could inhibit doxorubicin resistance in drug-resistant osteosarcoma cells [160]. The circRNA Hsa_circ_0004674 has been shown to increase the doxorubicin resistance of osteosarcoma cells by regulating the miR-342-3p/FBN1 axis [161].

### 9.2. Clinical Studies

The number of clinical studies investigating the potential of various ncRNAs as predictors of chemotherapy response in patients is significantly lower compared to the number of cell culture studies [162,163,164,165] (Table 4). In a general assessment, Chen et al. [166] concluded that drug resistance related miRNAs will probably supplement or may even partly replace existing biomarkers. In addition to this general assessment, several studies have been published in recent years that focus on specific microRNAs in this regard. For instance, the levels of miRNA-34a were measured in the serum of osteosarcoma patients with favorable and unfavorable responses to chemotherapy. Patients with histologically unfavorable responses exhibited significantly lower levels of that miRNA compared to patients with favorable responses [167]. The results by Diao et al. [168] revealed a significantly lower level of miRNA-22 in a group of 120 patients with highly malignant osteosarcoma. Low levels of miRNA-22 were significantly correlated with a poor tumor response to preoperative chemotherapy. In another study [169], it was confirmed that low serum levels of miRNA-375 were also significantly correlated with a poor tumor response to preoperative chemotherapy in 95 patients with highly malignant osteosarcoma, who graded the chemotherapy response according to the method by Huvos [170]. Moreover, miRNA-132 can be induced by angiogenic growth factors [171] and plays a role in the development of osteoarthritis [172]. Jie Yang et al. [173] analyzed Mi132 expression in the tissue of 166 osteosarcomas and the corresponding non-cancerous tissue. The miRNA-132 expression was found to be decreased in the osteosarcoma specimens with a poor response to chemotherapy. Yuan et al. [174] have demonstrated a correlation between high miRNA-21 levels and an advanced stage of disease, as defined by the Enneking classification. Furthermore, histological tumor response has been associated with an increased serum miRNA-21 level good in treatment responders compared to poor responders (*p* < 0.001). Another study also showed the usefulness of miRNA-21 for chemosensitivity prediction in osteosarcoma patients, with the miRNA-21 expression level of patients with osteosarcoma closely related to the therapeutic effects [175]. In an early study comprising 27 osteosarcoma patients, five miRNAs were identified, which can discriminate between a good and a poor chemotherapy response. MiR-92a, miR-99b, miR-193a-5p, and miR-422a were overexpressed in good chemotherapy responders, whereas miR-132 was downregulated [176].

In addition to miRNAs, circular RNAs have potential for predicting the chemotherapy response in osteosarcoma patients as well. The circular RNA, LARP4, showed a correlation with the histologically assessed response rate in 72 osteosarcoma patients after preoperative treatment with the MAP regimen (high-dose methotrexate, cisplatin, and doxorubicin). Patients with a good response to the treatment were Circ-LARP4 high and those with w low response were CircLARP4 low [177].

## 10. The ncRNAs and the Prediction of Metastatic Risk

It has been widely held that the conventional histological subtype of highly malignant osteosarcoma does not provide any discernible indicators of the likelihood of hematogenous metastasis development (Figure 5) [178]. But the structure of the extracellular matrix has been shown to contribute to metastasis and the progression of osteosarcoma [179]. Moreover, miRNAs are deeply involved in regulating angiogenesis, a central feature of metastasis, and the epithelial–mesenchymal transition. Because of these and other features, miRNAs have a high level of potential for use as biomarkers of metastatic risk [180,181]. A recent study has shown the potential of lncRNAs as prognostic biomarkers of metastatic colorectal cancer [182]. A very recent paper presents evidence that correlation changes in miRNAs with competing endogenous RNAs can predict whether and where metastases can occur in cancer patients at early stages [183]. In a similar way, lncRNAs are also deeply involved in the metastatic cascade. They contribute to the epithelial–mesenchymal transition, invasion, and migration, and are affiliated with nuclear factor κB and TGFβ pathways. Moreover, lncRNAs are useful indicators for assessing the metastatic risk in patients with different cancer entities, mostly carcinomas [184]. However, against this biologically promising background, clinical oncological studies performed on the serum/plasma or tumor tissue of osteosarcoma patients have been rather limited so far (Table 5), in comparison to cell culture studies, which have been performed abundantly [185].

A recent study by Abedi et al. [186] identified early diagnostic biomarkers, using miRNA expression profiles, associated with osteosarcoma metastasis. Based on network analysis and machine learning algorithms, new diagnostic tools have been established, which enable a reliable differentiation between metastatic osteosarcoma and non-metastatic samples, based on newly discovered miRNA signatures. The results showed that miR-34c-3p and miR-154-3p act as the most promising parameters in the diagnosis of metastatic osteosarcoma. In osteosarcoma, miRNAs and lncRNAs, as exosomal biomarkers, are predictors for the development of hematologic metastases [89]. Another study on exosomal biomarkers has shown that different miRNAs, such as miRNA-675, miRNA-1307, and miRNA-25-3p, and the lncRNAs, RAMP2-AS1 and CASC15, may be diagnostically useful for predicting metastatic risk in osteosarcoma and other sarcoma entities [187]. High levels of miRNA-34a in osteosarcoma patients not only correlate with the chemotherapy response, but also with longer overall survival and a decreased risk of metastasis as well [167]. Another miRNA with predictive potential for metastasis in osteosarcoma patients is miRNA-506, which revealed a significantly higher serum level in patients with non-metastatic osteosarcoma compared to patients with lung metastases [188]. These authors also suggest that an miRNA–mRNA network of higher complexity might in future serve as a predictive factor for hematogenic metastases in osteosarcoma. A study by Karras et al. (in preparation) investigating the differential miRNA expression between non-metastasizing primary osteosarcomas, primary osteosarcomas, and their lung and bone metastases, respectively, revealed the most differentially expressed miRNAs between the non-metastatic primary OS and the metastatic primary OS, particularly the metastatic primary OS that developed lung metastases. Further analysis is necessary to determine whether this result can be utilized as a predictor of metastatic potential in patients with primary osteosarcomas, who do not have hematogenic metastases at the time of the initial diagnosis.

## 11. Concluding Remarks

A histologic evaluation is still the most reliable and most effective method for diagnosing highly malignant osteosarcoma [1]. Despite well-established histologic diagnostics, highly malignant osteosarcoma can be misdiagnosed as another bone tumor, leading to catastrophic consequences, such as incorrect therapy and misguided surgical procedures [119]. Highly malignant osteosarcoma serves as a paradigmatic example of a tumor characterized by a high degree of molecular genetic complexity. This complexity is likely the primary reason why molecular genetic investigations have not yet yielded clinically significant diagnostic markers [14]. Therefore, additional methods are necessary to enhance differential diagnosis in this context. Given that over 98% of the human genome is non-coding, it is logical to explore diagnostic tools among the various types of ncRNAs [80,81,82]. Moreover, ncRNAs have demonstrated significant diagnostic potential in regard to tumors of other organs, particularly in distinguishing between benign and malignant tumors. However, their application as a diagnostic tool in bone tumor diagnosis has been limited so far, accounting for the focus of this review. To enhance the success of establishing ncRNAs as diagnostic tools in the field of osteosarcoma, more sophisticated deep cancer classifiers may be required [98]. This approach is anticipated to further reduce the incidence of misdiagnoses, based solely on histology, thereby ensuring the most effective treatment for bone tumor patients.

## Figures and Tables

**Table 1:** Examples of differentially expressed ncRNAs as diagnostic adjuncts for discriminating between benign and malignant lesions in several cancer entities.

Tumor Benign/Malignant	ncRNA	Material	Results	Source
Enchondroma/Chondrosarcoma	miR-181a and -138	Tumor tissue FFPE	Increased expression of miR-181a and -138 in low-grade chondrosarcoma compared with enchondroma	Zhang, L. et al., 2017 [110]
Benign Hyperplasia (BPH)/Prostatic Cancer	miR-27b-3p, miR-574-3p, miR-30a-5p, and miR-125b-5p	Urine	These miRNAs can be used to discriminate between BPH and prostatic cancer	Stella et al. [111]
Benign Nodules/Thyroid Cancer	miRNA-222	Serum	Discriminating between thyroid cancer and benign nodules	Bielak et al. [112]
High-risk Benign Breast Tumors/Breast Cancer	miRNAs, hsa-mir-128-3p, hsa-mir-421, hsa-mir-130b-5p, and hsa-mir-28-5p,	Plasma	Four miRNAs, hsa-mir-128-3p, hsa-mir-421, hsa-mir-130b-5p, and hsa-mir-28-5p, were differentially expressed in CA vs. HB, and had diagnostic power to discriminate CA from HB	Khadka et al. [114]
Benign Breast Disease/Breast Cancer	miR-106b-5p, -126-3p, -140-3p, -193a-5p, and -10b-5p	Plasma	Multi-marker panel consisting of hsa-miR-106b-5p, -126-3p, -140-3p, -193a-5p, and -10b-5p could detect the early stages of BC, with 0.79 sensitivity, 0.86 specificity, and 0.82 accuracy	Sadeghi et al. [113]
Benign Liver Tumors/Liver Cancer	LincRNA- 01093 lncRNA HELIS	Serum	LINC01093 and lncRNA HELIS are downregulated in all malignant liver cancers; in benign tumors, LINC01093 expression is only twice decreased in comparison to adjacent tissue samples	Burenina et al. [115]
Nonneoplastic Skin Diseases/Different Skin Cancers	miRNA-based deep cancer classifier miR-375 and miR-451	Serum	miR-375 and miR-451 are candidate biomarkers of neoplastic and non-neoplastic skin lesions	Kaczmarek et al. [98]
Benign and Malignant Effusions	miR-141-3p, miR-203a-3	Pleural fluid	Abundance of three miRNAs, miR-141-3p, miR-203a-3, and miR-200c-3p, correctly classifies malignant pleura effusions	Marques et al. [116]
Malignant Borderline Tumors/Ovarian Cancer	miR-30a-3p, miR-30c, miR-30d, and miR-30e-3p	Tumor tissue FFPE	Four miRNAs could discriminate mucinous borderline tumors from ovarian cancers	Dolivet et al. [117]
Benign Versus Malignant Adrenocortical Tumors	miR-139-3p, miR-335, miR-675		miRNA profiling of miR-675, miR-335, and miRNA-139-3p helps in discriminating ACCs from ACAs, adreno-cortical adenomas and carcinomas	Schmitz et al. [118]

**Table 2:** Examples of differentially expressed ncRNAs as diagnostic adjuncts in the differential diagnosis of highly malignant osteosarcoma.

Tumor Benign/Malignant	ncRNA	Material	Results	Source
Osteoblastoma/Osteosarcoma	miRNA-210	Tumor tissue FFPE	miRNA-210 displays low levels of expression across all of the osteoblastoma specimens and high expression in the majority of osteosarcoma specimens	Riester et al. [126]
Fibrous Dysplasia; Giant-Cell Tumor of Bone; Osteoblastoma; Chondrosarcoma Versus Osteosarcoma	miR-1261	Serum	Patients with osteosarcoma had higher serum miR-1261 levels than those with benign or intermediate-gradebone tumors	Araki Y et al., 2023 [43]

**Table 3:** The ncRNAs for early clinical diagnosis of osteosarcoma.

Non-Coding RNA	Materials	Results	Source
miR-1261	Serum	Higher miRNA serum levels point to a bone tumor of high-grade malignancy	Araki, A et al. [43]
miR-337-3p, miR-484, miR-582, miR-3677	Serum	These miRNAs were decreased in the serum of osteosarcoma patients	Luo, H et al. [132]
MiR-429 and MiR-143-3p	Serum	MiR-429 and miR-143-3p expression were significantly downregulated in the serum from OS patients	Yang, L et al. [133]
circRNA hsa_circ_0003074	Serum	hsa_circ_0003074 is highly expressed and is present in the peripheral blood of osteosarcoma patients	Lei, S et al. [134]
miR-101	Serum	miR-101 expression levels were under-expressed in serum samples from osteosarcoma patients compared to the controls	Yao, ZS et al. [135]
miR-124	Serum	The level of serum miR-124 was decreased in osteosarcoma patients when compared to healthy controls	Cong, C et al. [136]
miR-95-3p	Serum	Compared to healthy controls, the expression levels of miR-95-3p in the serum of osteosarcoma patients was significantly decreased	Niu, J et al. [137]
miRNA-223	Serum	The expression of miR-223 was significantly decreased in the serum of osteosarcoma patients compared to healthy controls	Dong, J et al. [138]
miR-195-5p, miR-199a-3p, miR-320a, and miR-374a-5p	Plasma	The expression levels were significantly increased in osteosarcoma patients and were markedly decreased in plasma after operation	Lian, F et al. [139]
microRNA-221	Serum; fresh frozen tissue	The expression levels of miR-221 in osteosarcoma tissues and sera were both upregulated	Yang, Z et al. [140]

**Table 4:** The ncRNAs from osteosarcoma patient’s serum, plasma, or sarcoma tissue, which have been identified as indicators of a poor response to chemotherapy.

Non-Coding RNA	Materials	Results	Source
miRNA-34a	Serum	Negatively associated with the chemotherapy resistance of OS patients	Lian, H. et al. [167]
miRNA-22	Plasma	Low plasma miR-22 levels were correlated with a poor tumor response to preoperative chemotherapy	Diao, ZB. et al. [168]
miRNA-375	Serum	A low serum miR-375 level was significantly associated with a poor tumor response to chemotherapy	Liu, W. et al. [169]
miRNA-132	Sarcoma tissue, fresh frozen	miR-132 expression was decreased in the osteosarcoma specimens from patients with a poor response to chemotherapy	Yang, J. et al. [173]
miRNA-21	Serum	High serum miR-21 was significantly correlated with an advanced Enneking stage and chemotherapeutic resistance	Yuan, J. et al. [174]
miRNA-21	Serum	The expression level of serum miR-21 in patients with osteosarcoma was closely related to the therapeutic effects of osteosarcoma	Hua, Y. et al. [175]
miR-92a, miR-99b, miR-132, miR-193a-5p, miR-422a	Sarcoma tissue, FFPE	The miRNAs, miR-92a, miR-99b, miR-132, miR-193a-5p, and miR-422a, could discriminate between good from bad responders	Gougelet, A. et al. [176]
circRNA LARP4	Sarcoma tissue, fresh frozen	The circ-LARP4 high-expression patients showed an increased tumor cell necrosis rate in response to adjuvant chemotherapy compared to the circ-LARP4 low-expression patients	Hu, Y. et al. [177]

**Table 5:** Non-coding RNAs with potential as predictors of hematogenic metastasis development in osteosarcoma patients.

Non-Coding RNA	Materials	Results	Source
miR-34c-3p and miR-154-3p	Sarcoma tissue, FFPE	The combined values of miR-34c-3p and miR-154-3p showed 90% diagnostic power for osteosarcoma samples and 85% for metastatic osteosarcoma	Abedi, S. et al. [186]
miR-675, miR-1307, miR-25-3p	Serum and plasma	Osteosarcoma-derived exosomal biomarkers, including miRNAs and lncRNAs, reveal diagnostic value and the potential to predict the prognosis for osteosarcoma metastasis	Tan, L. et al. [187]
miR-34a	Serum	Elevated serum levels of miR-34a were associated with a reduced incidence of metastasis in OS patients	Lian, H. et al. [167]
miR-506	Sarcoma tissue, FFPE	microRNA-506 was differentially expressed between osteosarcoma tissues with lung metastasis and non-metastatic tumor tissue	Meng, F. et al. [188]
miR-98-3p; miR-134-3p; miR-378C; miR-516A-5p; miR-548A-3p; miR-606; miR-650; miR-802; miR-1233-3p; miR-1271-3p; miR-3158-3p	Sarcoma tissue, FFPE	The most differentially expressed miRNAs (highly significant) were observed between the non-metastasizing OS and the metastasizing primary OS	Karras, F., in preparation

## References

[1] Yoshida A. (2021). Osteosarcoma: Old and New Challenges. Surg. Pathol. Clin..

[2] Savage S.A., Mirabello L. (2011). Using epidemiology and genomics to understand osteosarcoma etiology. Sarcoma.

[3] Nagano A., Matsumoto S., Kawai A., Okuma T., Hiraga H., Matsumoto Y., Nishida Y., Yonemoto T., Hosaka M., Takahashi M. (2020). Osteosarcoma in patients over 50 years of age: Multi-institutional retrospective analysis of 104 patients. J. Orthop. Sci..

[4] Baumhoer D., Brunner P., Eppenberger-Castori S., Smida J., Nathrath M., Jundt G. (2014). Osteosarcomas of the jaws differ from their peripheral counterparts and require a distinct treatment approach. Experiences from the DOESAK Registry. Oral Oncol..

[5] Robinson M.J., Davis E.J. (2024). Neoadjuvant Chemotherapy for Adults with Osteogenic Sarcoma. Curr. Treat. Options Oncol..

[6] Bielack S.S., Kempf-Bielack B., Delling G., Exner G.U., Flege S., Helmke K., Kotz R., Salzer-Kuntschik M., Werner M., Winkelmann W. (2002). Prognostic Factors in High-Grade Osteosarcoma of the Extremities or Trunk: An Analysis of 1702 Patients Treated on Neoadjuvant Cooperative Osteosarcoma Study Group Protocols. J. Clin. Oncol..

[7] Kager L., Zoubek A., Pötschger U., Kastner U., Flege S., Kempf-Bielack B., Branscheid D., Kotz R., Salzer-Kuntschik M., Winkelmann W. (2003). Primary Metastatic Osteosarcoma: Presentation and Outcome of Patients Treated on Neoadjuvant Cooperative Osteosarcoma Study Group Protocols. J. Clin. Oncol..

[8] Salzer-Kuntschik M., Delling G., Beron G., Sigmund R. (1983). Morphological grades of regression in osteosarcoma after polychemotherapy ? Study COSS 80. J. Cancer Res. Clin. Oncol..

[9] Bacci G., Bertoni F., Longhi A., Ferrari S., Forni C., Biagini R., Bacchini P., Donati D., Manfrini M., Bernini G. (2003). Neoadjuvant chemotherapy for high-grade central osteosarcoma of the extremity. Cancer.

[10] Green J.T., Mills A.M. (2014). Osteogenic tumors of bone. Semin Diagn. Pathol..

[11] Kansara M., Teng M.W., Smyth M.J., Thomas D.M. (2014). Translational biology of osteosarcoma. Nat. Rev. Cancer.

[12] Rickel K., Fang F., Tao J. (2017). Molecular genetics of osteosarcoma. Bone.

[13] Cè M., Cellina M., Ueanukul T., Carrafiello G., Manatrakul R., Tangkittithaworn P., Jaovisidha S., Fuangfa P., Resnick D. (2025). Multimodal Imaging of Osteosarcoma: From First Diagnosis to Radiomics. Cancers.

[14] Franceschini N., Lam S.W., Cleton-Jansen A.M., Bovée J.V.M.G. (2020). What’s new in bone forming tumours of the skeleton?. Virchows Archiv..

[15] Fan L., Zhong Z., Lin Y., Li J. (2022). Non-coding RNAs as potential biomarkers in osteosarcoma. Front. Genet..

[16] Mutsaers A.J., Walkley C.R. (2014). Cells of origin in osteosarcoma: Mesenchymal stem cells or osteoblast committed cells?. Bone.

[17] Klein M.J., Siegal G.P. (2006). Osteosarcoma: Anatomic and histologic variants. Am. J. Clin. Pathol..

[18] Chui M.H., Kandel R.A., Wong M., Griffin A.M., Bell R.S., Blackstein M.E., Wunder J.S., Dickson B.C. (2016). Histopathologic features of prognostic significance in high-grade osteosarcoma. Arch Pathol. Lab. Med..

[19] Masuda H., Miller C., Koeffler H.P., Battifora H., Cline M.J. (1987). Rearrangement of the p53 gene in human osteogenic sarcomas. Proc. Natl. Acad. Sci. USA.

[20] Toguchida J., Ishizaki K., Sasaki M.S., Nakamura Y., Ikenaga M., Kato M., Sugimot M., Kotoura Y., Yamamuro T. (1989). Preferential mutation of paternally derived RB gene as the initial event in sporadic osteosarcoma. Nature.

[21] Chen X., Bahrami A., Pappo A., Easton J., Dalton J., Hedlund E., Ellison D., Shurtleff S., Wu G., Wei L. (2014). Recurrent Somatic Structural Variations Contribute to Tumorigenesis in Pediatric Osteosarcoma. Cell Rep..

[22] Behjati S., Tarpey P.S., Haase K., Ye H., Young M.D., Alexandrov L.B., Farndon S.J., Collord G., Wedge D.C., Martincorena I. (2017). Recurrent mutation of IGF signalling genes and distinct patterns of genomic rearrangement in osteosarcoma. Nat. Commun..

[23] Bousquet M., Noirot C., Accadbled F., de Gauzy J.S., Castex M., Brousset P., Gomez-Brouchet A. (2016). Whole-exome sequencing in osteosarcoma reveals important heterogeneity of genetic alterations. Ann. Oncol..

[24] Chiappetta C., Mancini M., Lessi F., Aretini P., De Gregorio V., Puggioni C., Carletti R., Petrozza V., Civita P., Franceschi S. (2017). Whole-exome analysis in osteosarcoma to identify a personalized therapy. Oncotarget.

[25] Kovac M., Blattmann C., Ribi S., Smida J., Mueller N.S., Engert F., Castro-Giner F., Weischenfeldt J., Kovacova M., Krieg A. (2015). Exome sequencing of osteosarcoma reveals mutation signatures reminiscent of BRCA deficiency. Nat. Commun..

[26] Perry J.A., Kiezun A., Tonzi P., Van Allen E.M., Carter S.L., Baca S.C., Cowley G.S., Bhatt A.S., Rheinbay E., Pedamallu C.S. (2014). Complementary genomic approaches highlight the PI3K/mTOR pathway as a common vulnerability in osteosarcoma. Proc. Natl. Acad. Sci. USA.

[27] Jamshidi M., Pour S.M., Mahmoudian-Sani M.-R. (2020). Single nucleotide variants associated with colorectal cancer among iranian patients: A narrative review. Pharmacogenom. Pers. Med..

[28] Singh V., Katiyar A., Malik P., Kumar S., Mohan A., Singh H., Jain D. (2023). Identification of molecular biomarkers associated with non-small-cell lung carcinoma (NSCLC) using whole-exome sequencing. Cancer Biomark..

[29] Liguori L., Salomone F., Viggiano A., Sabbatino F., Pepe S., Formisano L., Bianco R., Servetto A. (2025). KRAS mutations in advanced non-small cell lung cancer: From biology to novel therapeutic strategies. Crit. Rev. Oncol. Hematol..

[30] Downing J.R., Wilson R.K., Zhang J., Mardis E.R., Pui C.-H., Ding L., Ley T.J., Evans W.E. (2013). The Pediatric Cancer Genome Project. Nat. Genet..

[31] Stephens P.J., Greenman C.D., Fu B., Yang F., Bignell G.R., Mudie L.J., Pleasance E.D., Lau K.W., Beare D., Stebbings L.A. (2011). Massive genomic rearrangement acquired in a single catastrophic event during cancer development. Cell.

[32] Nik-Zainal S., Alexandrov L.B., Wedge D.C., Van Loo P., Greenman C.D., Raine K., Jones D., Hinton J., Marshall J., Stebbings L.A. (2012). Mutational processes molding the genomes of 21 breast cancers. Cell.

[33] Nik-Zainal S., Davies H., Staaf J., Ramakrishna M., Glodzik D., Zou X., Martincorena I., Alexandrov L.B., Martin S., Wedge D.C. (2016). Landscape of somatic mutations in 560 breast cancer whole-genome sequences. Nature.

[34] Smida J., Baumhoer D., Rosemann M., Walch A., Bielack S., Poremba C., Remberger K., Korsching E., Scheurlen W., Dierkes C. (2010). Genomic alterations and allelic imbalances are strong prognostic predictors in osteosarcoma. Clin. Cancer Res..

[35] Amary M.F., Bacsi K., Maggiani F., Damato S., Halai D., Berisha F., Pollock R., O’Donnell P., Grigoriadis A., Diss T. (2011). IDH1 and IDH2 mutations are frequent events in central chondrosarcoma and central and periosteal chondromas but not in other mesenchymal tumours. J. Pathol..

[36] Suehara Y., Alex D., Bowman A., Middha S., Zehir A., Chakravarty D., Wang L., Jour G., Nafa K., Hayashi T. (2019). Clinical genomic sequencing of pediatric and adult osteosarcoma reveals distinct molecular subsets with potentially targetable alterations. Clin. Cancer Res..

[37] Baumhoer D., Hench J., Amary F. (2024). Recent advances in molecular profiling of bone and soft tissue tumors. Skelet. Radiol..

[38] Lam S.W., van Ijzendoorn D.G., Cleton-Jansen A.-M., Szuhai K., Bovée J.V. (2019). Molecular Pathology of Bone Tumors. J. Mol. Diagn..

[39] Baumhoer D., Amary F., Flanagan A.M. (2019). An update of molecular pathology of bone tumors. Lessons learned from investigating samples by next generation sequencing. Genes Chromosomes Cancer.

[40] Moonmuang S., Chaiyawat P., Jantrapirom S., Pruksakorn D., Piccolo L.L. (2021). Circulating long non-coding rnas as novel potential biomarkers for osteogenic sarcoma. Cancers.

[41] Dey M., Skipar P., Bartnik E., Piątkowski J., Sulejczak D., Czarnecka A.M. (2024). MicroRNA signatures in osteosarcoma: Diagnostic insights and therapeutic prospects. Mol. Cell. Biochem..

[42] Wang C., Ren M., Zhao X., Wang A., Wang J. (2018). Emerging roles of circular RNAs in osteosarcoma. Med. Sci. Monit..

[43] Araki Y., Asano N., Yamamoto N., Hayashi K., Takeuchi A., Miwa S., Igarashi K., Higuchi T., Abe K., Taniguchi Y. (2023). A validation study for the utility of serum microRNA as a diagnostic and prognostic marker in patients with osteosarcoma. Oncol. Lett..

[44] The ENCODE Project Consortium (2012). An integrated encyclopedia of DNA elements in the human genome. Nature.

[45] Beermann J., Piccoli M.-T., Viereck J., Thum T. (2016). Non-coding RNAs in Development and Disease: Background, Mechanisms, and Therapeutic Approaches. Physiol. Rev..

[46] Amaral P., Carbonell-Sala S., De La Vega F.M., Faial T., Frankish A., Gingeras T., Guigo R., Harrow J.L., Hatzigeorgiou A.G., Johnson R. (2023). The status of the human gene catalogue. Nature.

[47] Mattick J.S., Makunin I.V. (2006). Non-coding RNA. Hum. Mol. Genet..

[48] Srivastava M., Simakov O., Chapman J., Fahey B., Gauthier M.E.A., Mitros T., Richards G.S., Conaco C., Dacre M., Hellsten U. (2010). The Amphimedon queenslandica genome and the evolution of animal complexity. Nature.

[49] Slack F.J. (2006). Regulatory RNAs and the demise of “junk” DNA. Genome Biol..

[50] Willingham A.T., Gingeras T.R. (2006). TUF Love for “Junk” DNA. Cell.

[51] Ling H., Vincent K., Pichler M., Fodde R., Berindan-Neagoe I., Slack F.J., Calin G.A. (2015). Junk DNA and the long non-coding RNA twist in cancer genetics. Oncogene.

[52] Zuckerkandl E. (1992). Revisiting junk DNA. J. Mol. Evol..

[53] Mattick J.S. (2023). A Kuhnian revolution in molecular biology: Most genes in complex organisms express regulatory RNAs. BioEssays.

[54] Cech T.R., Steitz J.A. (2014). The noncoding RNA revolution—Trashing old rules to forge new ones. Cell.

[55] Mattick J.S. (2001). Non-coding RNAs: The architects of eukaryotic complexity. EMBO Rep..

[56] Yang J.X., Rastetter R.H., Wilhelm D. (2016). Non-coding RNAs: An introduction. Advances in Experimental Medicine and Biology.

[57] Taft R.J., Pheasant M., Mattick J.S. (2007). The relationship between non-protein-coding DNA and eukaryotic complexity. BioEssays.

[58] Morris K.V., Mattick J.S. (2014). The rise of regulatory RNA. Nat. Rev. Genet..

[59] Mattick J.S. (2011). The central role of RNA in human development and cognition. FEBS Lett..

[60] Gaiti F., Calcino A.D., Tanurdžić M., Degnan B.M. (2017). Origin and evolution of the metazoan non-coding regulatory genome. Dev. Biol..

[61] Graveley B.R. (2001). Alternative splicing: Increasing diversity in the proteomic world. TRENDS Genet..

[62] Nurk S., Koren S., Rhie A., Rautiainen M., Bzikadze A.V., Mikheenko A., Vollger M.R., Altemose N., Uralsky L., Gershman A. (2022). The complete sequence of a human genome. Science.

[63] Mattick J.S. (2023). RNA out of the mist. Trends Genet..

[64] Tétreault N., De Guire V. (2013). MiRNAs: Their discovery, biogenesis and mechanism of action. Clin. Biochem..

[65] Chung C.H., Bernard P.S., Perou C.M. (2002). Molecular portraits and the family tree of cancer. Nat. Genet..

[66] Bartel D.P. (2018). Metazoan MicroRNAs. Cell.

[67] Liu G., Mattick J.S., Taft R.J. (2013). A meta-analysis of the genomic and transcriptomic composition of complex life. Cell Cycle.

[68] Kim T., Croce C.M. (2023). MicroRNA: Trends in clinical trials of cancer diagnosis and therapy strategies. Exp. Mol. Med..

[69] Di Leva G., Croce C.M. (2013). MiRNA profiling of cancer. Curr. Opin. Genet. Dev..

[70] Qi P., Du X. (2013). The long non-coding RNAs, a new cancer diagnostic and therapeutic gold mine. Mod. Pathol..

[71] Ha M., Kim V.N. (2014). Regulation of microRNA biogenesis. Nat. Rev. Mol. Cell Biol..

[72] Castel S.E., Martienssen R.A. (2013). RNA interference in the nucleus: Roles for small RNAs in transcription, epigenetics and beyond. Nat. Rev. Genet.

[73] Cohen R.L., Settleman J. (2014). From cancer genomics to precision oncology—Tissue’s still an issue. Cell.

[74] Bianchi J.J., Zhao X., Mays J.C., Davoli T. (2020). Not all cancers are created equal: Tissue specificity in cancer genes and pathways. Curr. Opin. Cell Biol..

[75] Hoadley K.A., Yau C., Hinoue T., Wolf D.M., Lazar A.J., Drill E., Shen R., Taylor A.M., Cherniack A.D., Thorsson V. (2018). Cell-of-Origin Patterns Dominate the Molecular Article Cell-of-Origin Patterns Dominate the Molecular Classification of 10,000 Tumors from 33 Types of Cancer. Cell.

[76] Mansinho A., Fernandes R.M., Carneiro A.V. (2023). Histology-Agnostic Drugs: A Paradigm Shift—A Narrative Review. Adv. Ther..

[77] Schneider G., Schmidt-Supprian M., Rad R., Saur D. (2017). Tissue-specific tumorigenesis—Context matters. Nat. Rev. Cancer.

[78] Gaiti F., Fernandez-Valverde S.L., Nakanishi N., Calcino A.D., Yanai I., Tanurdzic M., Degnan B.M. (2015). Dynamic and widespread lncRNA expression in a sponge and the origin of animal complexity. Mol. Biol. Evol..

[79] Condrat C.E., Thompson D.C., Barbu M.G., Bugnar O.L., Boboc A., Cretoiu D., Suciu N., Cretoiu S.M., Voinea S.C. (2020). miRNAs as Biomarkers in Disease: Latest Findings Regarding Their Role in Diagnosis and Prognosis. Cells.

[80] Ludwig N., Leidinger P., Becker K., Backes C., Fehlmann T., Pallasch C., Rheinheimer S., Meder B., Stähler C., Meese E. (2016). Distribution of miRNA expression across human tissues. Nucleic Acids Res..

[81] Shademan B., Karamad V., Nourazarian A., Masjedi S., Isazadeh A., Sogutlu F., Avcı C.B. (2023). MicroRNAs as Targets for Cancer Diagnosis: Interests and Limitations. Adv. Pharm. Bull..

[82] Slack F.J., Chinnaiyan A.M. (2019). The Role of Non-coding RNAs in Oncology. Cell.

[83] Anastasiadou E., Jacob L.S., Slack F.J. (2017). Non-coding RNA networks in cancer. Nat. Rev. Cancer.

[84] Anastasiadou E., Faggioni A., Trivedi P., Slack F.J. (2018). The nefarious nexus of noncoding RNAs in cancer. Int. J. Mol. Sci..

[85] Wang H., Peng R., Wang J., Qin Z., Xue L. (2018). Circulating microRNAs as potential cancer biomarkers: The advantage and disadvantage. Clin. Epigenetics.

[86] Chen X., Liang H., Zhang J., Zen K., Zhang C.Y. (2012). Horizontal transfer of microRNAs: Molecular mechanisms and clinical applications. Protein Cell.

[87] Shyamala K., Girish H., Murgod S. (2014). Risk of tumor cell seeding through biopsy and aspiration cytology. J. Int. Soc. Prev. Community Dent..

[88] Kołat D., Hammouz R., Bednarek A.K., Płuciennik E. (2019). Exosomes as carriers transporting long non-coding RNAs: Molecular characteristics and their function in cancer. Mol. Med. Rep..

[89] Behulová R.L., Bugalová A., Bugala J., Struhárňanská E., Šafranek M., Juráš I. (2023). Circulating Exosomal miRNAs as a Promising Diagnostic Biomarker in Cancer. Physiol. Res..

[90] Chen M., Zhao H. (2019). Next-generation sequencing in liquid biopsy: Cancer screening and early detection. Hum. Genom..

[91] Kwapisz D. (2017). The first liquid biopsy test approved. Is it a new era of mutation testing for non-small cell lung cancer?. Ann. Transl. Med..

[92] Toden S., Goel A. (2022). Non-coding RNAs as liquid biopsy biomarkers in cancer. Br. J. Cancer.

[93] Ebrahimi N., Faghihkhorasani F., Fakhr S.S., Moghaddam P.R., Yazdani E., Kheradmand Z., Rezaei-Tazangi F., Adelian S., Mobarak H., Hamblin M.R. (2022). Tumor-derived exosomal non-coding RNAs as diagnostic biomarkers in cancer. Cell. Mol. Life Sci..

[94] Jordan-Alejandre E., Campos-Parra A.D., Castro-López D.L., Silva-Cázares M.B. (2023). Potential miRNA Use as a Biomarker: From Breast Cancer Diagnosis to Metastasis. Cells.

[95] Bartels C.L., Tsongalis G.J. (2009). MicroRNAs: Novel Biomarkers for Human Cancer CONTENT: Mini-Reviews. Clin. Chem..

[96] Lu J., Getz G., Miska E.A., Alvarez-Saavedra E., Lamb J., Peck D., Sweet-Cordero A., Ebert B.L., Mak R.H., Ferrando A.A. (2005). MicroRNA expression profiles classify human cancers. Nature.

[97] Crim J., Layfield L.J. (2023). Bone and soft tissue tumors at the borderlands of malignancy. Skelet. Radiol..

[98] Kaczmarek E., Pyman B., Nanayakkara J., Tuschl T., Tyryshkin K., Renwick N., Mousavi P. (2022). Discriminating Neoplastic from Nonneoplastic Tissues Using an miRNA-Based Deep Cancer Classifier. Am. J. Pathol..

[99] Kopp F., Mendell J.T. (2018). Functional Classification and Experimental Dissection of Long Noncoding RNAs. Cell.

[100] Mattick J.S. (2018). The state of long non-coding RNA biology. Noncoding RNA.

[101] Clark M.B., Mattick J.S. (2011). Long noncoding RNAs in cell biology. Semin. Cell Dev. Biol..

[102] Chi Y., Wang D., Wang J., Yu W., Yang J. (2019). Long non-coding RNA in the pathogenesis of cancers. Cells.

[103] Iyer M.K., Niknafs Y.S., Malik R., Singhal U., Sahu A., Hosono Y., Barrette T.R., Prensner J.R., Evans J.R., Zhao S. (2015). The landscape of long noncoding RNAs in the human transcriptome. Nat. Genet..

[104] Wei G., Zhu J., Hu H.B., Liu J.Q. (2021). Circular RNAs: Promising biomarkers for cancer diagnosis and prognosis. Gene.

[105] Pisignano G., Michael D.C., Visal T.H., Pirlog R., Ladomery M., Calin G.A. (2023). Going circular: History, present, and future of circRNAs in cancer. Oncogene.

[106] Zhang H., Shen Y., Li Z., Ruan Y., Li T., Xiao B., Sun W. (2020). The biogenesis and biological functions of circular RNAs and their molecular diagnostic values in cancers. J. Clin. Lab. Anal..

[107] Xian J., Su W., Liu L., Rao B., Lin M., Feng Y., Qiu F., Chen J., Zhou Q., Zhao Z. (2020). Identification of Three Circular RNA Cargoes in Serum Exosomes as Diagnostic Biomarkers of Non–Small-Cell Lung Cancer in the Chinese Population. J. Mol. Diagn..

[108] Pan B., Qin J., Liu X., He B., Wang X., Pan Y., Sun H., Xu T., Xu M., Chen X. (2019). Identification of Serum Exosomal hsa-circ-0004771 as a Novel Diagnostic Biomarker of Colorectal Cancer. Front. Genet..

[109] Su M., Xiao Y., Ma J., Tang Y., Tian B., Zhang Y., Li X., Wu Z., Yang D., Zhou Y. (2019). Circular RNAs in Cancer: Emerging functions in hallmarks, stemness, resistance and roles as potential biomarkers. Mol. Cancer.

[110] Zhang L., Yang M., Mayer T., Johnstone B., Les C., Frisch N., Parsons T., Mi Q.-S., Gibson G. (2017). Use of MicroRNA biomarkers to distinguish enchondroma from low-grade chondrosarcoma. Connect. Tissue Res..

[111] Stella M., Russo G.I., Leonardi R., Carcò D., Gattuso G., Falzone L., Ferrara C., Caponnetto A., Battaglia R., Libra M. (2024). Extracellular RNAs from Whole Urine to Distinguish Prostate Cancer from Benign Prostatic Hyperplasia. Int. J. Mol. Sci..

[112] Bielak C., Arya A., Savill S. (2023). Circulating microRNA as potential diagnostic and prognostic biomarkers of well-differentiated thyroid cancer: A review article. Cancer Biomark..

[113] Sadeghi H., Kamal A., Ahmadi M., Najafi H., Zarchi A.S., Haddad P., Shayestehpour B., Kamkar L., Salamati M., Geranpayeh L. (2021). A novel panel of blood-based microRNAs capable of discrimination between benign breast disease and breast cancer at early stages. RNA Biol..

[114] Khadka V.S., Nasu M., Deng Y., Jijiwa M. (2023). Circulating microRNA Biomarker for Detecting Breast Cancer in High-Risk Benign Breast Tumors. Int. J. Mol. Sci..

[115] Burenina O.Y., Lazarevich N.L., Kustova I.F., Shavochkina D.A., Moroz E.A., Kudashkin N.E., Patyutko Y.I., Metelin A.V., Kim E.F., Skvortsov D.A. (2021). Panel of potential lncRNA biomarkers can distinguish various types of liver malignant and benign tumors. J. Cancer Res. Clin. Oncol..

[116] Marqués M., Pont M., Hidalgo I., Sorolla M.A., Parisi E., Salud A., Sorolla A., Porcel J.M. (2023). MicroRNAs Present in Malignant Pleural Fluid Increase the Migration of Normal Mesothelial Cells In Vitro and May Help Discriminate between Benign and Malignant Effusions. Int. J. Mol. Sci..

[117] Dolivet E., Gaichies L., Jeanne C., Bazille C., Briand M., Vernon M., Giffard F., Leprêtre F., Poulain L., Denoyelle C. (2023). Synergy of the microRNA Ratio as a Promising Diagnosis Biomarker for Mucinous Borderline and Malignant Ovarian Tumors. Int. J. Mol. Sci..

[118] Schmitz K.J., Helwig J., Bertram S., Sheu S.Y., Suttorp A.C., Seggewiß J., Willscher E., Walz M.K., Worm K., Schmid K.W. (2011). Differential expression of microRNA-675, microRNA-139-3p and microRNA-335 in benign and malignant adrenocortical tumours. J. Clin. Pathol..

[119] Hecker-Nolting S., Baumhoer D., Blattmann C., Kager L., Kühne T., Kevric M., Lang S., Mettmann V., Sorg B., Werner M. (2023). Osteosarcoma pre-diagnosed as another tumor: A report from the Cooperative Osteosarcoma Study Group (COSS). J. Cancer Res. Clin. Oncol..

[120] Suster D., Mackinnon A.C., Jarzembowski J.A., Carrera G., Suster S., Klein M.J. (2022). Epithelioid osteoblastoma. Clinicopathologic and immunohistochemical study of 17 cases. Hum. Pathol..

[121] Gambarotti M., Tos A.P.D., Vanel D., Picci P., Gibertoni D., Klein M.J., Righi A. (2019). Osteoblastoma-like osteosarcoma: High-grade or low-grade osteosarcoma?. Histopathology.

[122] Fittall M.W., Mifsud W., Pillay N., Ye H., Strobl A.-C., Verfaillie A., Demeulemeester J., Zhang L., Berisha F., Tarabichi M. (2018). Recurrent rearrangements of FOS and FOSB define osteoblastoma. Nat. Commun..

[123] Lam S.W., Cleven A.H.G., Kroon H.M., Bruijn I.H.B.-D., Szuhai K., Bovée J.V.M.G. (2020). Utility of FOS as diagnostic marker for osteoid osteoma and osteoblastoma. Virchows Archiv..

[124] Amary F., Markert E., Berisha F., Ye H., Gerrand C., Cool P., Tirabosco R., Lindsay D., Pillay N., O’donnell P. (2019). FOS Expression in Osteoid Osteoma and Osteoblastoma: A Valuable Ancillary Diagnostic Tool. Am. J. Surg. Pathol..

[125] Ameline B., Nathrath M., Nord K.H., de Flon F.H., Bovée J.V., Krieg A.H., Höller S., Hench J., Baumhoer D. (2022). Methylation and copy number profiling: Emerging tools to differentiate osteoblastoma from malignant mimics?. Mod. Pathol..

[126] Riester S.M., Torres-Mora J., Dudakovic A., Camilleri E.T., Wang W., Xu F., Thaler R.R., Evans J.M., Zwartbol R., Bruijn I.H.B.-D. (2017). Hypoxia-related microRNA-210 is a diagnostic marker for discriminating osteoblastoma and osteosarcoma. J. Orthop. Res..

[127] Palmini G., Brandi M.L. (2021). microRNAs and bone tumours: Role of tiny molecules in the development and progression of chondrosarcoma, of giant cell tumour of bone and of Ewing’s sarcoma. Bone.

[128] Jiang P., Li Y., Yang X., Zhou J., Wei P. (2020). Clinical value of differential lncRNA expressions in diagnosis of giant cell tumor of bone and tumor recurrence. Clin. Lab..

[129] Qin S., He N.B., Yan H.L., Dong Y. (2016). Characterization of MicroRNA Expression Profiles in Patients with Giant Cell Tumor. Orthop. Surg..

[130] Lian K., Chen Z., Chen L., Li Y., Liu L. (2025). Network study of miRNA regulating traumatic heterotopic ossification. PLoS ONE.

[131] Mierzejewski B., Pulik Ł., Grabowska I., Sibilska A., Ciemerych M.A., Łęgosz P., Brzoska E. (2023). Coding and noncoding RNA profile of human heterotopic ossifications—Risk factors and biomarkers. Bone.

[132] Luo H., Ye Z. (2021). Identification of serum miR-337-3p, miR-484, miR-582, and miR-3677 as promising biomarkers for osteosarcoma. Clin. Lab..

[133] Yang L., Li H., Huang A. (2020). MiR-429 and MiR-143-3p function as diagnostic and prognostic markers for osteosarcoma. Clin. Lab..

[134] Lei S., Xiang L. (2020). Up-regulation of circrna hsa_circ_0003074 expression is a reliable diagnostic and prognostic biomarker in patients with osteosarcoma. Cancer Manag. Res..

[135] Yao Z.-S., Li C., Liang D., Jiang X.-B., Tang J.-J., Ye L.-Q., Yuan K., Ren H., Yang Z.-D., Jin D.-X. (2018). Diagnostic and prognostic implications of serum miR-101 in osteosarcoma. Cancer Biomark..

[136] Cong C., Wang W., Tian J., Gao T., Zheng W., Zhou C. (2018). Identification of serum miR-124 as a biomarker for diagnosis and prognosis in osteosarcoma. Cancer Biomark..

[137] Niu J., Sun Y., Guo Q., Niu D., Liu B. (2016). Serum miR-95-3p is a diagnostic and prognostic marker for osteosarcoma. Springerplus.

[138] Dong J., Liu Y., Liao W., Liu R., Shi P., Wang L. (2016). MiRNA-223 is a potential diagnostic and prognostic marker for osteosarcoma. J. Bone Oncol..

[139] Lian F., Cui Y., Zhou C., Gao K., Wu L. (2015). Identification of a plasma four-microRNA panel as potential noninvasive biomarker for osteosarcoma. PLoS ONE.

[140] Yang Z., Zhang Y., Zhang X., Zhang M., Liu H., Zhang S., Qi B., Sun X. (2015). Serum microRNA-221 functions as a potential diagnostic and prognostic marker for patients with osteosarcoma. Biomed. Pharmacother..

[141] Rosen G., Caparros B., Huvos A.G., Kosloff C., Nirenberg A., Cacavio A., Marcove R.C., Lane J.M., Mehta B., Urban C. (1982). Preoperative chemotherapy for osteogenic sarcoma: Selection of postoperative adjuvant chemotherapy based on the response of the primary tumor to preoperative chemotherapy. Cancer.

[142] Glasser D.B., Lane J.M., Huvos A.G., Marcove R.C., Rosen G. (1992). Survival, prognosis, and therapeutic response in osteogenic sarcoma. The memorial hospital experience. Cancer.

[143] Davis A.M., Bell R.S., Goodwin P.J. (1994). Prognostic factors in osteosarcoma: A critical review. J. Clin. Oncol..

[144] O’kane G.M., Cadoo K.A., Walsh E.M., Emerson R., Dervan P., O’keane C., Hurson B., O’toole G., Dudeney S., Kavanagh E. (2015). Perioperative chemotherapy in the treatment of osteosarcoma: A 26-year single institution review. Clin. Sarcoma Res..

[145] Ho D.J., Agaram N.P., Jean M.-H., Suser S.D., Chu C., Vanderbilt C.M., Meyers P.A., Wexler L.H., Healey J.H., Fuchs T.J. (2023). Deep Learning–Based Objective and Reproducible Osteosarcoma Chemotherapy Response Assessment and Outcome Prediction. Am. J. Pathol..

[146] Byun B.H., Kong C.-B., Lim I., Kim B.I., Choi C.W., Song W.S., Cho W.H., Jeon D.-G., Koh J.-S., Lee S.-Y. (2014). Early response monitoring to neoadjuvant chemotherapy in osteosarcoma using sequential 18F-FDG PET/CT and MRI. Eur. J. Nucl. Med. Mol. Imaging.

[147] Miwa S., Takeuchi A., Shirai T., Taki J., Yamamoto N., Nishida H., Hayashi K., Tanzawa Y., Kimura H., Igarashi K. (2013). Prognostic Value of Radiological Response to Chemotherapy in Patients with Osteosarcoma. PLoS ONE.

[148] Laux C.J., Berzaczy G., Weber M., Lang S., Dominkus M., Windhager R., Nöbauer-Huhmann I.-M., Funovics P.T. (2015). Tumour response of osteosarcoma to neoadjuvant chemotherapy evaluated by magnetic resonance imaging as prognostic factor for outcome. Int. Orthop..

[149] Cheng W., Zhang Z., Deng S., Yang C. (2021). Comprehensive Analysis of Key mRNAs and lncRNAs in Osteosarcoma Response to Preoperative Chemotherapy with Prognostic Values. Curr. Med. Sci..

[150] Carrle D., Bielack S.S. (2006). Current strategies of chemotherapy in osteosarcoma. Int. Orthop..

[151] Tsukamoto S., Errani C., Angelini A., Mavrogenis A.F. (2020). Current treatment considerations for osteosarcoma metastatic at presentation. Orthopedics.

[152] Lin Z., Xie X., Lu S., Liu T. (2021). Noncoding RNAs in osteosarcoma: Implications for drug resistance. Cancer Lett..

[153] Xu W., Li Z., Zhu X., Xu R., Xu Y. (2018). MiR-29 family inhibits resistance to methotrexate and promotes cell apoptosis by targeting COL3A1 and MCL1 in osteosarcoma. Med. Sci. Monit..

[154] Li G., Zhu Y. (2019). Effect of lncRNA ANRIL knockdown on proliferation and cisplatin chemoresistance of osteosarcoma cells in vitro. Pathol. Res. Pract..

[155] Li G., Yan X. (2023). Long non-coding RNA GAS5 promotes cisplatin-chemosensitivity of osteosarcoma cells via microRNA-26b-5p/TP53INP1 axis. J. Orthop. Surg. Res..

[156] Dong L., Qu F. (2020). CircUBAP2 promotes SEMA6D expression to enhance the cisplatin resistance in osteosarcoma through sponging miR-506-3p by activating Wnt/β-catenin signaling pathway. J. Mol. Histol..

[157] Zhang Z., Zhou Q., Luo F., Zhou R., Xu J., Xiao J., Dai F., Song L. (2021). Circular RNA circ-CHI3L1.2 modulates cisplatin resistance of osteosarcoma cells via the miR-340-5p/LPAATβ axis. Hum. Cell.

[158] Guo J., Dou D., Zhang T., Wang B. (2020). HOTAIR Promotes Cisplatin Resistance of Osteosarcoma Cells by Regulating Cell Proliferation, Invasion, and Apoptosis via miR-106a-5p/STAT3 Axis. Cell Transpl..

[159] Ling Z., Fan G., Yao D., Zhao J., Zhou Y., Feng J., Zhou G., Chen Y. (2020). MicroRNA-150 functions as a tumor suppressor and sensitizes osteosarcoma to doxorubicin-induced apoptosis by targeting RUNX2. Exp. Ther. Med..

[160] Wang X., Ding R., Fu Z., Yang M., Li D., Zhou Y., Qin C., Zhang W., Si L., Zhang J. (2024). Overexpression of miR-506-3p reversed doxorubicin resistance in drug-resistant osteosarcoma cells. Front. Pharmacol..

[161] Bai Y., Li Y., Bai J., Zhang Y. (2021). Hsa_circ_0004674 promotes osteosarcoma doxorubicin resistance by regulating the miR-342-3p/FBN1 axis. J. Orthop. Surg. Res..

[162] Pei Y., Li S. (2025). Unraveling the impact of noncoding RNAs in osteosarcoma drug resistance: A review of mechanisms and therapeutic implications. Int. J. Surg..

[163] He H., Ni J., Huang J. (2014). Molecular mechanisms of chemoresistance in osteosarcoma (review). Oncol. Lett..

[164] Hu S., Han X., Liu G., Wang S. (2024). LncRNAs as potential prognosis/diagnosis markers and factors driving drug resistance of osteosarcoma, a review. Front. Endocrinol..

[165] Chen H., Gong Z., Zhou H., Han Y. (2024). Deciphering chemoresistance in osteosarcoma: Unveiling regulatory mechanisms and function through the lens of noncoding RNA. Drug Dev. Res..

[166] Chen R., Wang G., Zheng Y., Hua Y., Cai Z. (2019). Drug resistance-related microRNAs in osteosarcoma: Translating basic evidence into therapeutic strategies. J. Cell. Mol. Med..

[167] Lian H., Zhou Y., Sun Z., Liu K. (2022). MicroRNA34a is associated with chemotherapy resistance, metastasis, recurrence, survival, and prognosis in patient with osteosarcoma. Medicine.

[168] Diao Z.B., Sun T.X., Zong Y., Lin B.C., Xia Y.S. (2020). Identification of plasma microRNA-22 as a marker for the diagnosis, prognosis, and chemosensitivity prediction of osteosarcoma. J. Int. Med. Res..

[169] Liu W., Zhao X.T., Zhang Y.J., Fang G.W., Xue Y. (2018). MicroRNA-375 as a potential serum biomarker for the diagnosis, prognosis, and chemosensitivity prediction of osteosarcoma. J. Int. Med. Res..

[170] Rosen G., Murphy M.L., Huvos A.G., Gutierrez M., Marcove R.C. (1976). Chemotherapy, en bloc resection, and prosthetic bone replacement in the treatment of osteogenic sarcoma. Cancer.

[171] Anand S., Majeti B.K., Acevedo L.M., Murphy E.A., Mukthavaram R., Scheppke L., Huang M., Shields D.J., Lindquist J.N., Lapinski P.E. (2010). MicroRNA-132-mediated loss of p120RasGAP activates the endothelium to facilitate pathological angiogenesis. Nat. Med..

[172] Zhang W., Hu C., Zhang C., Luo C., Zhong B., Yu X. (2021). MiRNA-132 regulates the development of osteoarthritis in correlation with the modulation of PTEN/PI3K/AKT signaling. BMC Geriatr..

[173] Yang J., Gao T., Tang J., Cai H., Lin L., Fu S. (2013). Loss of microRNA-132 predicts poor prognosis in patients with primary osteosarcoma. Mol. Cell. Biochem..

[174] Yuan J., Chen L., Chen X., Sun W., Zhou A.X. (2012). Identification of Serum MicroRNA-21 as a Biomarker for Chemosensitivity and Prognosis in Human Osteosarcoma. J. Int. Med. Res..

[175] Hua Y., Jin Z., Zhou F., Zhang Y.-Q., Zhuang Y. (2017). Serum MiR-21 function in osteosarcoma chemosensitivity. Eur. Rev. Med. Pharmacol. Sci..

[176] Gougelet A., Pissaloux D., Besse A., Perez J., Duc A., Dutour A., Blay J., Alberti L. (2011). Micro-RNA profiles in osteosarcoma as a predictive tool for ifosfamide response. Int. J. Cancer.

[177] Hu Y., Gu J., Shen H., Shao T., Li S., Wang W., Yu Z. (2020). Circular RNA LARP4 correlates with decreased Enneking stage, better histological response, and prolonged survival profiles, and it elevates chemosensitivity to cisplatin and doxorubicin via sponging microRNA-424 in osteosarcoma. J. Clin. Lab. Anal..

[178] Roessner A., Lohmann C., Jechorek D. (2021). Translational cell biology of highly malignant osteosarcoma. Pathol. Int..

[179] Cui J., Dean D., Hornicek F.J., Chen Z., Duan Z. (2020). The role of extracelluar matrix in osteosarcoma progression and metastasis. J. Exp. Clin. Cancer Res..

[180] Grzywa T.M., Klicka K., Włodarski P.K. (2020). Regulators at every step—How microRNAs drive tumor cell invasiveness and metastasis. Cancers.

[181] Baldasici O., Pileczki V., Cruceriu D., Gavrilas L.I., Tudoran O., Balacescu L., Vlase L., Balacescu O. (2022). Breast Cancer-Delivered Exosomal miRNA as Liquid Biopsy Biomarkers for Metastasis Prediction: A Focus on Translational Research with Clinical Applicability. Int. J. Mol. Sci..

[182] Kenari S.N., Mohamadynejad P., Moghanibashi M., Bagheri A., Rouhi L. (2025). Upregulation of LncRNAs G2E3-AS1 and BACE1-AS as prognostic biomarkers in metastatic colorectal cancer. Biomarkers.

[183] Cho M., Park B., Han K. (2025). Predicting distant metastatic sites of cancer using perturbed correlations of miRNAs with competing endogenous RNAs. Comput. Biol. Chem..

[184] Liu S.J., Dang H.X., Lim D.A., Feng F.Y., Maher C.A. (2021). Long noncoding RNAs in cancer metastasis. Nat. Rev. Cancer.

[185] Mosca N., Alessio N., Di Paola A., Marrapodi M.M., Galderisi U., Russo A., Rossi F., Potenza N. (2024). Osteosarcoma in a ceRNET perspective. J. Biomed. Sci..

[186] Abedi S., Behmanesh A., Mazhar F.N., Bagherifard A., Sami S.H., Heidari N., Hossein-Khannazer N., Namazifard S., Arki M.K., Shams R. (2024). Machine learning and experimental analyses identified miRNA expression models associated with metastatic osteosarcoma. Biochim. Biophys. Acta Mol. Basis Dis..

[187] Tan L., Wang Y., Hu X., Min L. (2023). The Roles of Exosomes in Metastasis of Sarcoma: From Biomarkers to Therapeutic Targets. Biomolecules.

[188] Meng F., Wang L., Gao G., Chen J., Wang X., Wu G., Miu Y. (2022). Identification and verification of microRNA signature and key genes in the development of osteosarcoma with lung metastasis. Medicine.

